# Association of Optical Coherence Tomography–Measured Fibrovascular Ridge Thickness and Clinical Disease Stage in Retinopathy of Prematurity

**DOI:** 10.1001/jamaophthalmol.2022.4173

**Published:** 2022-10-13

**Authors:** Thanh-Tin P. Nguyen, Shuibin Ni, Susan Ostmo, Archeta Rajagopalan, Aaron S. Coyner, Mani Woodward, Michael F. Chiang, Yali Jia, David Huang, J. Peter Campbell, Yifan Jian

**Affiliations:** 1 Casey Eye Institute, Oregon Health & Science University, Portland; 2 Department of Biomedical Engineering, Oregon Health & Science University, Portland; 3 School of Medicine, University of Southern California, Los Angeles; 4 National Eye Institute, National Institutes of Health, Bethesda, Maryland

## Abstract

**Importance:**

Accurate diagnosis of retinopathy of prematurity (ROP) is essential to provide timely treatment and reduce the risk of blindness. However, the components of an ROP examination are subjective and qualitative.

**Objective:**

To evaluate whether optical coherence tomography (OCT)–derived retinal thickness measurements at the vascular-avascular junction are associated with clinical diagnosis of ROP stage.

**Design, Setting, and Participants:**

This cross-sectional longitudinal study compared OCT-based ridge thickness calculated from OCT B-scans by a masked examiner to the clinical diagnosis of 2 masked examiners using both traditional stage classifications and a more granular continuous scale at the neonatal intensive care unit (NICU) of Oregon Health & Science University (OHSU) Hospital. Infants who met ROP screening criteria in the OHSU NICU between June 2021 and April 2022 and had guardian consent were included. One OCT volume and en face image per patient per eye showing at least 1 to 2 clock hours of ridge were included in the final analysis.

**Main Outcomes and Measures:**

Comparison of OCT-derived ridge thickness to the clinical diagnosis of ROP stage using an ordinal and continuous scale. Repeatability was assessed using 20 repeated examinations from the same visit and compared using intraclass correlation coefficient (ICC) and coefficient of variation (CV). Comparison of ridge thickness with ordinal categories was performed using generalized estimating equations and with continuous stage using Spearman correlation.

**Results:**

A total of 128 separate OCT eye examinations from 50 eyes of 25 patients were analyzed. The ICC was 0.87 with a CV of 7.0%. Higher ordinal disease classification was associated with higher axial ridge thickness on OCT, with mean (SD) thickness measurements of 264.2 (11.2) μm (*P* < .001), 334.2 (11.4) μm (*P* < .001), and 495.0 (32.2) μm (*P* < .001) for stages 1, 2, and 3, respectively and with continuous stage labels (ρ = 0.739, *P* < .001).

**Conclusions and Relevance:**

These results suggest that OCT-based quantification of peripheral stage in ROP may be an objective and quantitative biomarker that may be useful for clinical diagnosis and longitudinal monitoring and may have implications for disease classification in the future.

## Key Points

**Question:**

What is the association between clinical disease stage on en face imaging and the axial fibrovascular ridge thickness in retinopathy of prematurity (ROP) as measured by swept-source optical coherence tomography (OCT)?

**Findings:**

In this cross-sectional study of 50 eyes from 25 patients, OCT measurements of ridge thickness were associated with an increase in higher clinical classification of ROP disease stage.

**Meaning:**

The findings indicate that fibrovascular ridge thickness may be a quantifiable biomarker for ROP diagnosis and useful for longitudinal monitoring of ROP disease progression.

## Teaser

This cross-sectional study evaluates the association between the clinical diagnosis of retinopathy of prematurity and optical coherence tomography–measured fibrovascular ridge thickness.

## Introduction

The diagnosis of retinopathy of prematurity (ROP) is guided by the International Classification of ROP (ICROP), which was updated in 2021 in part to reflect advances in understanding of ROP diagnosis provided by modern ophthalmic imaging devices.^1^ Using either ophthalmoscopic examination or fundus photography with telemedical or image-based interpretation, clinicians are responsible for classifying the zone of disease (how much of the retina is vascularized), the stage of disease (the degree of abnormal vascularization at the vascular-avascular junction), and the presence of plus disease (the dilation and tortuosity of retinal blood vessels). Accurate diagnosis of zone, stage, and plus are essential because these determine evidence-based intervention strategies.

Despite the presence of standard photographs for instruction and comparison, there is widespread evidence that clinical diagnoses exhibit interobserver variability for all components of the ICROP classification. This is due to the challenges of the ophthalmoscopic examination, related in part to geographic and training differences in clinical diagnosis, but also because continuous features (ie, zone, stage, and plus) are classified into ordinal disease categories, such as normal, pre-plus, or plus. Recent work evaluating clinical classification from expert members of the ICROP 3 committee^2^ revealed that clinical diagnosis of both plus and stage appeared to reflect a spectrum of pathology, with good clinical agreement at the ends of the spectrum, but disagreement in between. Artificial intelligence–based analysis of fundus photographs represents one potential method of providing objective assessment of disease severity of both stage and plus disease^2,3,4^ and may play a role in ROP screening in the future.

Optical coherence tomography can provide not only en face visualization of the retinal surface, but also volumetric data that facilitate objective diagnosis with higher resolution than the clinical examination. As a result, OCT has changed not only the way many adult retinal diseases are diagnosed, but also the existing disease classification systems and treatment paradigms. There have been 2 main limitations preventing the same benefits from being applied to pediatric retinal diseases such as ROP: imaging speed and narrow field of view (FOV) of available devices. However, recent advances in swept-source handheld OCT devices have been promising.^5,6,7,8^ Previous work has provided qualitative evidence that OCT B-scans reveal progressive changes in the retinal topography associated with increasing disease stage.^9,10,11,12^

There are several approaches to visualize the retinal periphery, including the use of scleral depression and optical design changes that improve the FOV at the expense of image resolution.^10,11^ In collaboration with the Center for Ophthalmic Optics and Lasers (COOL) Lab at Oregon Health & University, we recently described a portable ultra-widefield swept-source , and OCT device with a handheld probe^13,14^ that can reliably obtain views of the peripheral stage in ROP.^15,16,17^ In this article, we evaluate whether the retinal thickness as measured on B-scans at the vascular-avascular junction may provide a quantifiable biomarker of ROP stage.

## Methods

### OCT Imaging Protocol and Data Set Selection

This study was approved by the institutional review board at Oregon Health & Science University and adheres to all tenets of the Declaration of Helsinki. Written consent for imaging was obtained from guardians of all infants included in the study. Study participants did not receive any stipends or incentives to participate. Ultra-widefield–OCT images were acquired from all infants whose guardians gave consent and who met ROP screening criteria (birth weight ≤1500 g; gestational age ≤30 weeks) at Oregon Health & Science University Hospital between June 2021 and April 2022. Images were captured with a 400-kHz portable handheld swept-source OCT system after pharmacological dilation and placement of an eyelid speculum. The prototype OCT device uses a modular lens system that allows both noncontact 105° FOV imaging and 140° FOV with a contact approach. Switching between the 2 FOV systems does not impact retinal tissue thickness measurement in the axial direction, as the fundamental OCT engine (light source, detector, and digitizer) does not change. This device has an imaging range of 6 mm in air, which translates to 4.5 mm in tissue (4.4 μm per pixel in the axial dimension). In general, multiple scans of each eye were obtained for each examination. En face OCT images from each volume were reviewed to identify the highest-quality scan for segmentation. Images were chosen if they showed at least 1 to 2 clock hours of peripheral stage. For the primary analysis, imaging sessions were excluded if there was no peripheral stage present (ie, stage 0 ROP) on the en face OCT images or if the patient had previously received treatment. In a secondary analysis, we analyzed ridge thickness longitudinally in patients pretreatment and posttreatment with intravitreal bevacizumab.

### Clinical Diagnosis of ROP Stage

Two masked graders, 1 practicing ROP clinician and researcher (J.P.C.) and 1 study coordinator and ROP program manager with extensive experience in ROP image-based diagnosis (S.O.), were asked to assign stage classifications to each mean intensity en face OCT image using both ordinal stage (eg, 1, 2, 3) as well as continuous stage labels (with 0.1 increments). The continuous labels from the 2 graders were then averaged. Disagreement in ordinal labels was adjudicated by rounding continuous labels to the nearest integer. This produced 2 sets of stage classification for each image: ordinal (consistent with ICROP) and a more continuous label (consistent with the spectrum of expert diagnosis of stage).^2^ Images were presented to each reader in a randomized order with uniform contrast across all en face images. Images with a mean stage label of less than 0.5 were not analyzed further due to there being no perceptible ridge to trace at the vascular-avascular junction. We compared the overall agreement and weighted κ statistic for ordinal class gradings and the Pearson correlation coefficient for continuous gradings between J.P.C. and S.O.

### OCT Image Analysis

**Figure 1.**
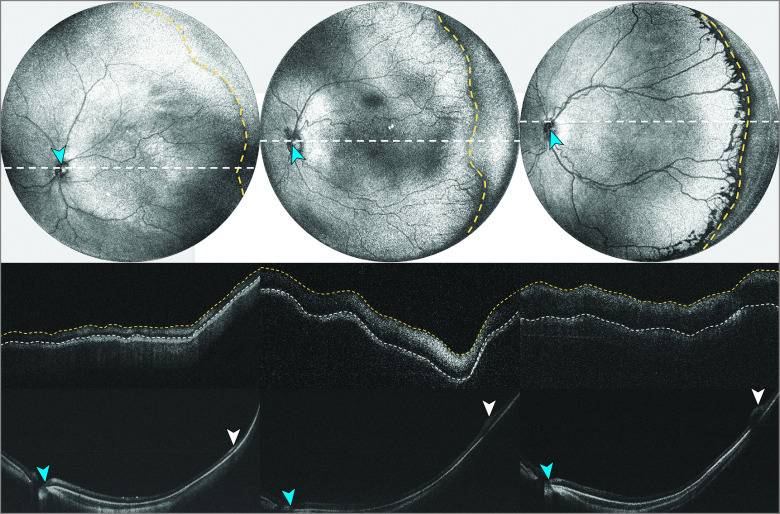
Methodology for Assessing Ridge Thickness Optical coherence tomography (top row) en face with manual tracing of the fibrovascular ridge (yellow dotted line) with resultant B-scans (middle row) manually segmented between the retinal pigment epithelium (white dotted line) and inner ridge surface to determine thickness. B-scans (bottom row) taken from the optic nerve (blue arrowheads) to the ridge. The peak of the ridge is denoted by white arrowheads. From left to right stage 1, 2, and 3.

OCT volumes were processed and presented in linear scale. A graphical user interface developed in-house using MATLAB version 2021B (MathWorks, Inc) was used to visualize the OCT en face. The peak of the ridgeline was then manually traced and segmented along the vascular-avascular junction on the vascular side (Figure 1). The user could toggle between mean and maximum intensity projections, adjust the number of adjacent B-scans registered and averaged, and adjust contrast for en face and B-scan images to identify the optimal tracing. Segmentation and manual ridge tracings were performed in a masked fashion relative to demographic and clinical information. On the B-scan, the area between the retinal pigment epithelium and the inner surface of the ridge (retinal or fibrovascular ridge vs vitreous interface) was segmented. Thickness was calculated pixelwise in the axial dimension and the top 10 maximum thickness measurements along the ridge were averaged to calculate maximum thickness for the ridge (presented as means with SDs). Ordinal stage was compared to maximum thickness using a generalized estimating equation to account for intereye and interexamination correlations. The association between continuous stage and maximum thickness was assessed via Spearman correlation.

### Repeatability

We included a repeatability analysis of ridgeline tracings produced using our method. We analyzed ridge thickness measurements of 2 captures of the same portion of the ridge during the same session. We analyzed 20 eyes (40 total measurements) with multiple scans from the same visit and analyzed the precision using an intraclass correlation and mean coefficient of variation across the 20 eyes. Volumes used for precision measurements were selected at random among all eyes with repeat intravisit imaging.

### Posttreatment Subgroup Analysis

We performed a separate analysis on infants treated with intravitreal bevacizumab. The maximum ridge thickness was compared 1 to 2 weeks before treatment, at the time of treatment, and 1 to 2 weeks after treatment. The generalized estimating equation was used for this analysis.

### Statistical Analysis

All statistical analyses were performed using R version 4.2.1 (R Foundation). The tests run were Pearson correlation, Spearman correlation, Kohen weighted κ, and generalized estimating equations. All tests were 2-tailed and significance was defined as *P* < .05.

## Results

### Demographic Characteristics

The final data set included 128 OCT imaging sessions from 50 eyes of 25 patients. Eight eyes from 4 infants were imaged over the course of 37 distinct imaging sessions, which were used for analysis of pretreatment and posttreatment thickness. Intergrader agreement on ordinal stage labels was 87% (weighted κ, 0.54) with a Pearson correlation coefficient of 0.76 for continuous labels (Table).

**Table.** Patient Demographic Characteristics

Characteristic	Main analysis group	Pretreatment and posttreatment group
No. of patients	25	4
No. of eyes	50	8
Female	15 (60.0)	2 (50.0)
Male	10 (40.0)	2 (50.0)
Gestational age, mean (SD), wk	26.7 (2.4)	24.6 (1.1)
Birth weight, mean (SD), g	834.3 (350.3)	495.3 (86.8)
Postmenstrual age, mean (SD), wk	37.3 (3.7)	36.7 (1.7)
No. of OCT volumes	128	24
OCT scan stage diagnosis		
Stage 1	50	0^a^
Stage 2	65	7
Stage 3	13	1

Abbreviation: OCT, optical coherence tomography.

^a^ Stage at time of treatment.

### Axial Ridge Thickness by Clinical Disease Stage

**Figure 2.**
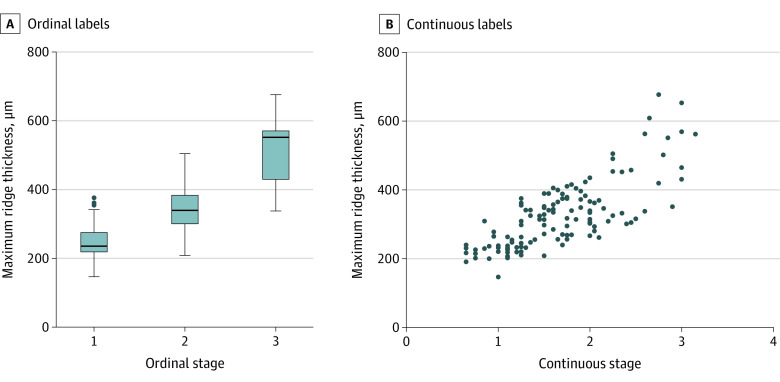
Axial Thickness of Fibrovascular Ridge by Ordinal and Continuous Stage Labels A, Higher ridge thickness on optical coherence tomography was associated with higher ordinal stage labels from masked graders based on en face optical coherence tomography appearance (*P* < .001). B, The association between ridge thickness and continuous stage label appeared to be linear (Spearman ρ = 0.739; *P* < .001).

Higher ordinal disease classification was associated with higher axial ridge thickness on OCT, with stages 1, 2, and 3 ROP having mean (SD) maximum ridge thickness estimates equal to 264.2 (11.2) μm (*P* < .001), 334.2 (11.4) μm (*P* < .001), and 495.0 (32.2) μm (*P* < .001), respectively, as determined by generalized estimating equation (Figure 2A). Similarly, the Spearman rank correlation between maximum ridge thickness and continuous stage labels was 0.739 (*P* < .001, Figure 2B). Intravisit repeatability was high, as assessed by intraclass correlation (0.87) and coefficient of variation (7.0%).

### Posttreatment Subgroup Analysis

**Figure 3.**
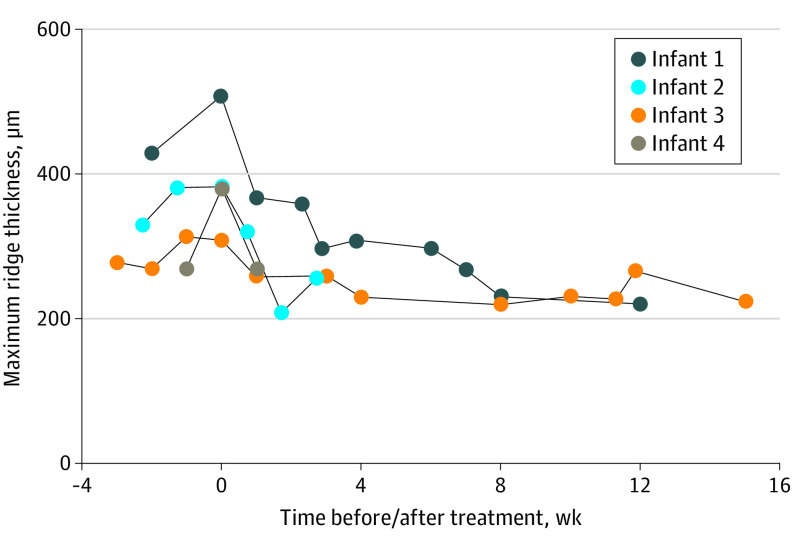
Longitudinal Analysis of Ridge Thickness Pretreatment and Posttreatment for Retinopathy of Prematurity Longitudinal trends in maximum ridge thickness pretreatment and posttreatment in 4 patients treated with intravitreal bevacizumab. Right and left eyes were all treated at the same time (time = 0 weeks), and results are the mean of right and left eye values for each infant.

During the study period, 4 infants were diagnosed with type 1 ROP and treated with intravitreal bevacizumab. Mean (SE) maximum ridge thickness was 342.4 (32.5) μm 1 to 2 weeks prior to treatment (*P* < .001), increasing to 394.4 (22.3) μm at time of treatment (*P* = .02) and decreasing to 304.0 (12.6) μm 1 to 2 weeks posttreatment *(P* *=* .002). Figure 3 shows the progression of all 4 infants over time, pretreatment and posttreatment.

## Discussion

Previous work has shown that spectral-domain OCT can produce B-scans that qualitatively differ between clinical disease stages and correspond to what is known from histology.^9^ In this study, we report quantitative assessment of maximal ridge thickness compared with masked clinical assessment of stage from en face OCT images using both the traditional ordinal scale and a continuous scale. There were 3 key findings. First, increased ridge thickness was associated with an increase in ordinal disease stage. Second, a continuous stage label was correlated with ridge thickness, highlighting the spectrum of ROP stage. Third, although this observation was limited by small numbers, we observed a rapid decrease in ridge thickness following treatment with bevacizumab.

Quantification of the thickness of peripheral pathology at the vascular-avascular junction (disease stage) using OCT may provide an objective marker of disease severity that correlates with clinical diagnosis. Previous work evaluating interobserver agreement in assessment of ROP stage on fundus photographs has shown patterns both of temporal drift (changing diagnosis over time for the same image by groups of people) and bias as to the level of pathology necessary to classify stage into ordinal categories.^1,2^ This is similar to what has been observed previously with plus disease.^1,2,18^ Along with assessment of vascular severity using a more granular scale for plus disease, or using artificial intelligence, these results suggest that objective evaluation using OCT may represent another method of quantifying stage severity in ROP in the future.

**Figure 4.**
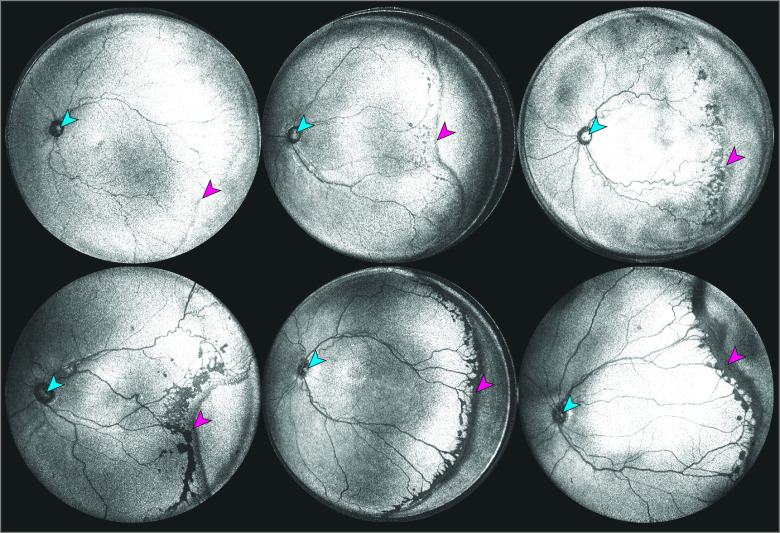
Spectrum of Pathology at the Vascular-Avascular Border in Retinopathy of Prematurity as Visualized on En Face Ultra-Widefield Optical Coherence Tomography Scans Six representative en face optical coherence tomography scans demonstrating a range of peripheral pathology both in degree and extent. Blue arrowheads indicate the optic nerve heads and pink arrowheads indicate the fibrovascular ridge.

These results further suggest that diagnosis of stage using a more granular scale is both feasible and more consistent with measurements of ridge thickness than current ordinal stage classifications. Further, these results only scratch the surface as to the precision of OCT-based stage assessment since ROP stage varies in axial thickness not only at 1 location, but for 360°, as shown in Figure 4. This highlights another potential limitation of our current classification system, which records only the maximum stage in the eye regardless of extent, which may oversimplify the spectrum of disease. Careful clinical observations of pathology at the vascular-avascular junction, such as those by Chen et al^9^ may reveal new insights into ROP progression and regression patterns, and the spectrum of retinoschisis and retinal detachment.^9^ Using spectral-domain OCT, Chen et al observed that the ridge terminated slightly anterior to the vascular-avascular junction in stage 2 ROP. Additionally, they found that splitting of retinal layers in stage 3 ROP occurred on either side of neovascularization, and there was an absence of preretinal structures (ie, popcorn neovascularization) over the ridge itself, with noteworthy thickening immediately along the avascular side of the ridge. Li et al^19^ quantified the dimensions and vascularization of the ridge in the transverse dimension using automated segmentation of en face fundus photographs and found a significant difference in ridge width and vascular proliferation for each stage. Future work incorporating volumetric visualization may further add to our understanding of the associations between overall retinal thickness and disease stage and severity and may lead to changes in ROP disease classification, as OCT has for other common retinal diseases.^20^

OCT-based longitudinal assessment of both ridge thickness and overall retinal and choroidal volumes may further enhance our understanding of ROP physiology. Previous work using an artificial intelligence–based vascular severity score compared with clinical diagnosis has suggested that changes in vascular severity (dilation and tortuosity) are associated with changes in the stage and/or extent of disease. Using OCT to visualize the retinal periphery, it is possible to observe clinical progression of so-called popcorn retinal neovascularization, which occurs posterior to the ridge, as well as the coalescing into the typical stage 3 lesion, the development of retinoschisis and tractional retinal detachment, and the relationship between those features and vascular severity in the posterior retina.^10,11,12^ In addition, OCT may be useful for monitoring and characterizing patterns of disease regression following both spontaneous resolution of disease and treatment-induced disease regression.^9^

### Limitations

There are several limitations to this study. The study device is a clinical research prototype and not commercially available. The process of measuring ridge thickness in this article was performed manually, although it could be automated in the future. Correlation with disease labels is also limited by interobserver variability, which we tried to mitigate using 2 masked graders. We did not address issues of image quality, since we manually chose the best en face images from each examination, though we had fairly high repeatability and were able to obtain views of the retinal periphery in all images with study imaging performed. Additionally, due to the clinical need to keep examinations of neonates brief and reduce neonatal stress as much as possible, we did not directly compare with the ophthalmoscopic examination with scleral depression.

## Conclusions

In this cross-sectional study, OCT-based measurement of peripheral ridge thickness correlated with image-based stage diagnosis and highlighted the spectrum of vascular pathology in ROP. While not yet widely available, these results suggest that OCT may one day be used for ultra-widefield anatomic staging of ROP, more precisely characterizing the degree and extent of peripheral pathology. OCT is also superior to the ophthalmoscopic examination for identifying early vitreoretinal traction, which means surgical intervention could be timed early to prevent macula involving retinal detachments. Finally, OCT may contribute to refinements in ROP disease classification in the future.
